# *N*-(2,5-Dimeth­oxy­phen­yl)-*N*′-[4-(2-hy­droxy­eth­yl)phen­yl]urea

**DOI:** 10.1107/S1600536810033520

**Published:** 2010-08-25

**Authors:** Hyeong Choi, Yong Suk Shim, Byung Hee Han, Sung Kwon Kang, Chang Keun Sung

**Affiliations:** a Department of Chemistry, Chungnam National University, Daejeon 305-764, Republic of Korea; b Department of Food Science and Technology, Chungnam National University, Daejeon 305-764, Republic of Korea

## Abstract

In the title compound, C_17_H_20_N_2_O_4_, the 2,5-dimeth­oxy­phenyl unit is essentially planar, with an r.m.s. deviation of 0.015 Å. The dihedral angle between the benzene rings is 43.66 (4)°. The mol­ecular structure is stabilized by a short intra­molecular N—H⋯O hydrogen bond. In the crystal, inter­molecular N—H⋯O and O—H⋯O hydrogen bonds link the mol­ecules into a three-dimensional network.

## Related literature

For general background to melanin synthesis, melanogenesis and tyrosinase, see: Francisco *et al.* (2006 ▶); Hearing & Jimenez (1987 ▶); Prota (1988 ▶); Grimes *et al.* (2006 ▶); Maeda & Fukuda (1991 ▶). For the development of potent inhibitory agents of tyrosinase and melanin formation as whitening agents, see: Ohguchi *et al.* (2003 ▶); Lemic-Stojcevic *et al.* (1995 ▶); Battaini *et al.* (2000 ▶); Cabanes *et al.* (1994 ▶); Liangli (2003 ▶); Thanigaimalai *et al.* (2010 ▶); Hong *et al.* (2008 ▶); Lee *et al.* (2007 ▶); Yi *et al.* (2009 ▶, 2010 ▶); Kwak *et al.* (2010 ▶); Choi *et al.* (2010 ▶); Germanas *et al.* (2007 ▶); Briganti *et al.* (2003 ▶).
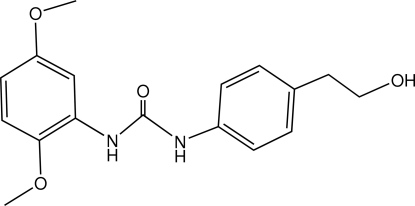


## Experimental

### 

#### Crystal data

C_17_H_20_N_2_O_4_
*M* *_r_* = 316.35
Monoclinic, 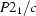

*a* = 18.7551 (18) Å
*b* = 6.8095 (6) Å
*c* = 12.6881 (12) Å
β = 98.930 (3)°
*V* = 1600.8 (3) Å^3^
*Z* = 4
Mo *K*α radiation
μ = 0.09 mm^−1^
*T* = 296 K
0.31 × 0.28 × 0.08 mm

#### Data collection

Bruker SMART CCD area-detector diffractometer
13398 measured reflections
3563 independent reflections
2473 reflections with *I* > 2σ(*I*)
*R*_int_ = 0.045

#### Refinement

*R*[*F* ^2^ > 2σ(*F* ^2^)] = 0.038
*wR*(*F* ^2^) = 0.108
*S* = 1.03
3563 reflections
222 parameters
H atoms treated by a mixture of independent and constrained refinement
Δρ_max_ = 0.15 e Å^−3^
Δρ_min_ = −0.16 e Å^−3^

### 

Data collection: *SMART* (Bruker, 2002 ▶); cell refinement: *SAINT* (Bruker, 2002 ▶); data reduction: *SAINT*; program(s) used to solve structure: *SHELXS97* (Sheldrick, 2008 ▶); program(s) used to refine structure: *SHELXL97* (Sheldrick, 2008 ▶); molecular graphics: *DIAMOND* (Brandenburg, 2010 ▶); software used to prepare material for publication: *WinGX* (Farrugia, 1999 ▶).

## Supplementary Material

Crystal structure: contains datablocks global, I. DOI: 10.1107/S1600536810033520/vm2041sup1.cif

Structure factors: contains datablocks I. DOI: 10.1107/S1600536810033520/vm2041Isup2.hkl

Additional supplementary materials:  crystallographic information; 3D view; checkCIF report

## Figures and Tables

**Table 1:** Hydrogen-bond geometry (Å, °)

*D*—H⋯*A*	*D*—H	H⋯*A*	*D*⋯*A*	*D*—H⋯*A*
N7—H7⋯O20	0.865 (15)	2.227 (14)	2.6113 (16)	106.7 (11)
O19—H19⋯O9^i^	0.867 (18)	1.841 (19)	2.7080 (14)	178.0 (17)
N10—H10⋯O19^ii^	0.867 (14)	2.161 (14)	2.9799 (15)	157.4 (12)
N7—H7⋯O19^ii^	0.865 (15)	2.189 (15)	2.9837 (15)	152.6 (13)

Symmetry codes: (i) 
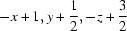
; (ii) 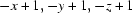.

**Fig. 1.**
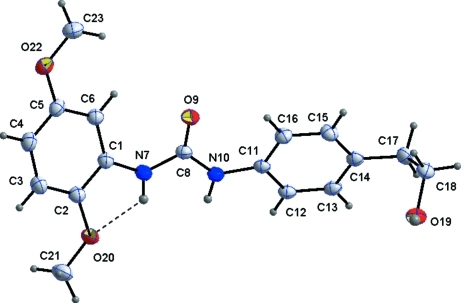
Molecular structure of (I), showing the atom-numbering scheme and 50% probability ellipsoids. Intramolecular N—H···O bond is shown as dashed lines.

**Fig. 2.**
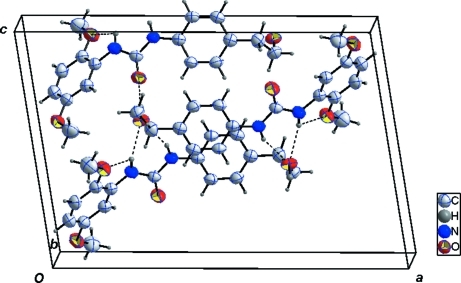
Part of the crystal structure of (I), showing 3-D network of molecules linked by intermolecular N—H···O and O—H···O hydrogen bonds.

## supplementary crystallographic information

### Comment

Melanin synthesis is the major source of skin color and plays an important role in protection against ultraviolet rays from the sun (Francisco *et al.*, 2006). Melanogenesis is initiated with the first step of tyrosine oxidation to dopaquinone catalyzed by tyrosinase (Hearing & Jimenez, 1987). Tyrosinase known as a polyphenol oxidase (PPO), is a multifunctional copper-containing enzyme widely distributed in nature, including bacteria, fungi, higher plants and animals (Prota, 1988). However, overproduction of melanin posed not only an esthetic problem but also a dermatological issue (Grimes *et al.*, 2006). Therefore, tyrosinase inhibitors have become increasingly important for medicinal, food and cosmetic products that may be used to prevent or treat pigmentation disorders (Maeda & Fukuda, 1991). In this regard, diverse tyrosinase inhibitors have been actively discovered such as hydroxystilbene compounds like resveratrol (Ohguchi *et al.*, 2003), azelaic acid (Lemic-Stojcevic *et al.*, 1995), kojic acid (Battaini *et al.*, 2000), albutin (Cabanes *et al.*, 1994), (*R*)-HTCCA (Liangli, 2003) and *N*-phenylthiourea (Thanigaimalai *et al.*, 2010). They contain aromatic, methoxy, hydroxyl (Hong *et al.*, 2008; Lee *et al.*, 2007), aldehyde (Yi *et al.*, 2010), amide (Kwak *et al.*, 2010; Choi *et al.*, 2010), thiosemicarbazone (Yi *et al.*, 2009) and thiazole (Germanas *et al.*, 2007) groups in their structure, and act as a specific functional group to make the skin whiter by inhibiting the production of melanin. Although numerous compounds have been reported as skin whitening depigmenting agents, most of them have been utilized for the treatment of hyperpigmentation disorders, but none are completely satisfactory owing to adverse effects such as toxicity and/or safety concerns (Briganti *et al.*, 2003). In our continuing search for tyrosinase inhibitors, we have synthesized the title compound, (I), from the reaction of 4-aminophenethyl alcohol and 2,5-dimethoxyphenyl isocyanate under ambient condition. Here, the crystal structure of (I) is described (Fig.1).

The 2,5-dimethoxyphenyl moiety is essentially planar, with r.m.s. deviation of 0.015 Å from the corresponding least-squares plane defined by the eleven constituent atoms. The dihedral angle between the benzene rings is 43.66 (4) °. The molecular structure is stabilized by a short intramolecular N7—H7···O20 hydrogen bond (Fig. 1). In the crystal, intermolecular N—H···O and O—H···O hydrogen bonds link the molecules into a three-dimensional network (Fig. 2, Table 1).

### Experimental

4-aminophenethyl alcohol and 2,5-dimethoxyphenyl isocyanate were purchased from Sigma Chemical Co. Solvents used for organic synthesis were redistilled before use. All other chemicals and solvents were of analytical grade and were used without further purification. The title compound (I) was prepared from the reaction of 4-aminophenethyl alcohol (0.18 g, 1.2 mmol) with 2,5-dimethoxyphenyl isocyanate (0.2 g, 1 mmol) in acetonitrile (6 ml). The reaction was completed within 30 min at room temperature. The reaction mixture was filtered, the solids collected and washed with dried hexane. Removal of the solvent gave a white solid (90%, m.p. 427 K). Single crystals were obtained by slow evaporation in ethanol at room temperature.

### Refinement

The H atoms of the NH and OH groups were located in a difference Fourier map and refined freely. The remaining H atoms were positioned geometrically and refined using a riding model with C—H = 0.93–0.97 Å, and with *U*_iso_(H) = 1.2*U*_eq_ (C) for aromatic and metylene, and 1.5*U*_eq_(C) for methyl H atoms.

### Crystal data

**Table:**

C_17_H_20_N_2_O_4_	*F*(000) = 672
*M**_r_* = 316.35	*D*_x_ = 1.313 Mg m^−^^3^
Monoclinic, *P*2_1_/*c*	Mo *K*α radiation, λ = 0.71073 Å
Hall symbol: -P 2ybc	Cell parameters from 4670 reflections
*a* = 18.7551 (18) Å	θ = 2.9–28.0°
*b* = 6.8095 (6) Å	µ = 0.09 mm^−^^1^
*c* = 12.6881 (12) Å	*T* = 296 K
β = 98.930 (3)°	Plate, colourless
*V* = 1600.8 (3) Å^3^	0.31 × 0.28 × 0.08 mm
*Z* = 4	

### Data collection

**Table:**

Bruker SMART CCD area-detector diffractometer	*R*_int_ = 0.045
φ and ω scans	θ_max_ = 27.5°, θ_min_ = 2.2°
13398 measured reflections	*h* = −20→24
3563 independent reflections	*k* = −8→5
2473 reflections with *I* > 2σ(*I*)	*l* = −16→7

### Refinement

**Table:**

Refinement on *F*^2^	0 restraints
Least-squares matrix: full	H atoms treated by a mixture of independent and constrained refinement
*R*[*F*^2^ > 2σ(*F*^2^)] = 0.038	*w* = 1/[σ^2^(*F*_o_^2^) + (0.0527*P*)^2^ + 0.087*P*] where *P* = (*F*_o_^2^ + 2*F*_c_^2^)/3
*wR*(*F*^2^) = 0.108	(Δ/σ)_max_ < 0.001
*S* = 1.03	Δρ_max_ = 0.15 e Å^−^^3^
3563 reflections	Δρ_min_ = −0.16 e Å^−^^3^
222 parameters	

### Special details

**Table:**

Geometry. All e.s.d.'s (except the e.s.d. in the dihedral angle between two l.s. planes) are estimated using the full covariance matrix. The cell e.s.d.'s are taken into account individually in the estimation of e.s.d.'s in distances, angles and torsion angles; correlations between e.s.d.'s in cell parameters are only used when they are defined by crystal symmetry. An approximate (isotropic) treatment of cell e.s.d.'s is used for estimating e.s.d.'s involving l.s. planes.

### Fractional atomic coordinates and isotropic or equivalent isotropic displacement parameters (Å^2^)

**Table:**

	*x*	*y*	*z*	*U*_iso_*/*U*_eq_	
C1	0.81571 (7)	0.4546 (2)	0.70224 (10)	0.0478 (3)	
C2	0.85245 (8)	0.6263 (2)	0.68225 (10)	0.0512 (3)	
C3	0.91672 (8)	0.6722 (2)	0.74533 (12)	0.0594 (4)	
H3	0.9415	0.7852	0.7315	0.071*	
C4	0.94471 (8)	0.5516 (2)	0.82909 (12)	0.0603 (4)	
H4	0.9877	0.5848	0.8723	0.072*	
C5	0.90884 (8)	0.3820 (2)	0.84865 (11)	0.0533 (3)	
C6	0.84471 (8)	0.3320 (2)	0.78530 (11)	0.0515 (3)	
H6	0.8211	0.2166	0.7983	0.062*	
N7	0.75121 (7)	0.41428 (19)	0.63308 (9)	0.0557 (3)	
H7	0.7438 (8)	0.482 (2)	0.5747 (12)	0.061 (4)*	
C8	0.69226 (7)	0.31819 (18)	0.65892 (10)	0.0456 (3)	
O9	0.69222 (6)	0.23814 (17)	0.74516 (8)	0.0696 (3)	
N10	0.63478 (6)	0.32245 (17)	0.58002 (9)	0.0483 (3)	
H10	0.6403 (7)	0.3846 (19)	0.5222 (11)	0.049 (4)*	
C11	0.56284 (7)	0.26392 (17)	0.58430 (10)	0.0420 (3)	
C12	0.51483 (8)	0.26886 (18)	0.48941 (10)	0.0461 (3)	
H12	0.5314	0.3014	0.4263	0.055*	
C13	0.44293 (8)	0.22611 (18)	0.48762 (11)	0.0475 (3)	
H13	0.4118	0.2295	0.423	0.057*	
C14	0.41582 (7)	0.17787 (17)	0.58034 (11)	0.0460 (3)	
C15	0.46477 (8)	0.1691 (2)	0.67378 (11)	0.0542 (4)	
H15	0.4483	0.134	0.7366	0.065*	
C16	0.53723 (8)	0.2105 (2)	0.67713 (10)	0.0519 (3)	
H16	0.5687	0.2026	0.7413	0.062*	
C17	0.33652 (8)	0.1382 (2)	0.57897 (12)	0.0567 (4)	
H17A	0.3309	0.0088	0.6086	0.068*	
H17B	0.3124	0.1373	0.5056	0.068*	
C18	0.30012 (9)	0.2874 (2)	0.64108 (12)	0.0585 (4)	
H18A	0.2503	0.2494	0.6416	0.07*	
H18B	0.3245	0.2921	0.7143	0.07*	
O19	0.30252 (6)	0.47658 (15)	0.59305 (8)	0.0582 (3)	
H19	0.3042 (9)	0.563 (3)	0.6438 (15)	0.085 (6)*	
O20	0.81964 (6)	0.73557 (16)	0.59742 (9)	0.0684 (3)	
C21	0.85404 (10)	0.9101 (3)	0.57235 (14)	0.0816 (5)	
H21A	0.8596	0.9967	0.6328	0.122*	
H21B	0.8253	0.9731	0.5127	0.122*	
H21C	0.9007	0.8792	0.5545	0.122*	
O22	0.94077 (6)	0.27125 (18)	0.93408 (9)	0.0717 (3)	
C23	0.90471 (10)	0.1000 (3)	0.96033 (14)	0.0786 (5)	
H23A	0.8569	0.1336	0.9725	0.118*	
H23B	0.931	0.0422	1.0237	0.118*	
H23C	0.9017	0.0077	0.9026	0.118*	

### Atomic displacement parameters (Å^2^)

**Table:**

	*U*^11^	*U*^22^	*U*^33^	*U*^12^	*U*^13^	*U*^23^
C1	0.0455 (8)	0.0566 (8)	0.0415 (7)	−0.0014 (6)	0.0072 (6)	−0.0008 (6)
C2	0.0520 (9)	0.0562 (8)	0.0455 (7)	−0.0027 (7)	0.0075 (7)	0.0017 (6)
C3	0.0537 (9)	0.0628 (9)	0.0619 (9)	−0.0110 (7)	0.0099 (8)	−0.0016 (7)
C4	0.0474 (9)	0.0749 (10)	0.0567 (9)	−0.0025 (7)	0.0017 (7)	−0.0022 (8)
C5	0.0476 (8)	0.0672 (9)	0.0445 (7)	0.0075 (7)	0.0051 (6)	0.0014 (7)
C6	0.0512 (9)	0.0565 (8)	0.0468 (7)	−0.0010 (6)	0.0073 (7)	0.0019 (6)
N7	0.0557 (8)	0.0655 (8)	0.0434 (6)	−0.0115 (6)	−0.0001 (6)	0.0129 (6)
C8	0.0517 (8)	0.0435 (7)	0.0408 (7)	0.0000 (6)	0.0049 (6)	0.0041 (5)
O9	0.0631 (7)	0.0879 (8)	0.0550 (6)	−0.0102 (5)	0.0000 (5)	0.0303 (6)
N10	0.0527 (7)	0.0535 (6)	0.0381 (6)	−0.0037 (5)	0.0048 (5)	0.0070 (5)
C11	0.0501 (8)	0.0355 (6)	0.0400 (6)	0.0001 (5)	0.0053 (6)	−0.0010 (5)
C12	0.0579 (9)	0.0431 (7)	0.0368 (6)	0.0010 (6)	0.0062 (6)	0.0032 (5)
C13	0.0566 (9)	0.0420 (6)	0.0409 (7)	0.0016 (6)	−0.0017 (6)	−0.0002 (5)
C14	0.0530 (8)	0.0362 (6)	0.0482 (7)	−0.0004 (6)	0.0057 (6)	−0.0019 (5)
C15	0.0608 (10)	0.0614 (8)	0.0416 (7)	−0.0048 (7)	0.0118 (7)	0.0031 (6)
C16	0.0558 (9)	0.0613 (8)	0.0369 (7)	−0.0025 (7)	0.0012 (6)	0.0023 (6)
C17	0.0572 (9)	0.0500 (7)	0.0623 (9)	−0.0067 (7)	0.0070 (7)	0.0022 (7)
C18	0.0607 (10)	0.0674 (9)	0.0497 (8)	−0.0025 (7)	0.0154 (7)	0.0092 (7)
O19	0.0772 (7)	0.0569 (6)	0.0417 (5)	0.0039 (5)	0.0133 (5)	−0.0018 (5)
O20	0.0651 (7)	0.0690 (7)	0.0666 (7)	−0.0169 (5)	−0.0045 (6)	0.0212 (5)
C21	0.0899 (13)	0.0691 (10)	0.0816 (12)	−0.0219 (9)	0.0003 (10)	0.0213 (9)
O22	0.0582 (7)	0.0874 (8)	0.0647 (7)	0.0025 (6)	−0.0060 (5)	0.0186 (6)
C23	0.0776 (12)	0.0815 (12)	0.0741 (11)	0.0069 (9)	0.0035 (9)	0.0232 (10)

### Geometric parameters (Å, °)

**Table:**

C1—C6	1.3875 (19)	C13—C14	1.3913 (18)
C1—C2	1.4000 (19)	C13—H13	0.93
C1—N7	1.4069 (18)	C14—C15	1.3835 (19)
C2—O20	1.3731 (17)	C14—C17	1.5090 (19)
C2—C3	1.375 (2)	C15—C16	1.382 (2)
C3—C4	1.381 (2)	C15—H15	0.93
C3—H3	0.93	C16—H16	0.93
C4—C5	1.378 (2)	C17—C18	1.512 (2)
C4—H4	0.93	C17—H17A	0.97
C5—O22	1.3787 (17)	C17—H17B	0.97
C5—C6	1.381 (2)	C18—O19	1.4286 (17)
C6—H6	0.93	C18—H18A	0.97
N7—C8	1.3677 (17)	C18—H18B	0.97
N7—H7	0.865 (15)	O19—H19	0.867 (18)
C8—O9	1.2226 (15)	O20—C21	1.4122 (18)
C8—N10	1.3528 (17)	C21—H21A	0.96
N10—C11	1.4159 (17)	C21—H21B	0.96
N10—H10	0.867 (14)	C21—H21C	0.96
C11—C12	1.3874 (19)	O22—C23	1.414 (2)
C11—C16	1.3878 (18)	C23—H23A	0.96
C12—C13	1.3764 (19)	C23—H23B	0.96
C12—H12	0.93	C23—H23C	0.96
			
C6—C1—C2	119.60 (13)	C15—C14—C13	116.99 (13)
C6—C1—N7	123.65 (13)	C15—C14—C17	121.59 (12)
C2—C1—N7	116.72 (12)	C13—C14—C17	121.42 (13)
O20—C2—C3	125.36 (13)	C16—C15—C14	122.41 (12)
O20—C2—C1	114.92 (12)	C16—C15—H15	118.8
C3—C2—C1	119.72 (13)	C14—C15—H15	118.8
C2—C3—C4	120.47 (14)	C15—C16—C11	119.71 (13)
C2—C3—H3	119.8	C15—C16—H16	120.1
C4—C3—H3	119.8	C11—C16—H16	120.1
C5—C4—C3	119.93 (14)	C14—C17—C18	113.43 (12)
C5—C4—H4	120	C14—C17—H17A	108.9
C3—C4—H4	120	C18—C17—H17A	108.9
C4—C5—O22	115.81 (13)	C14—C17—H17B	108.9
C4—C5—C6	120.46 (14)	C18—C17—H17B	108.9
O22—C5—C6	123.73 (14)	H17A—C17—H17B	107.7
C5—C6—C1	119.80 (13)	O19—C18—C17	109.69 (11)
C5—C6—H6	120.1	O19—C18—H18A	109.7
C1—C6—H6	120.1	C17—C18—H18A	109.7
C8—N7—C1	126.35 (11)	O19—C18—H18B	109.7
C8—N7—H7	115.6 (10)	C17—C18—H18B	109.7
C1—N7—H7	116.0 (10)	H18A—C18—H18B	108.2
O9—C8—N10	124.10 (13)	C18—O19—H19	107.0 (12)
O9—C8—N7	122.73 (13)	C2—O20—C21	117.91 (12)
N10—C8—N7	113.16 (11)	O20—C21—H21A	109.5
C8—N10—C11	128.33 (11)	O20—C21—H21B	109.5
C8—N10—H10	116.8 (9)	H21A—C21—H21B	109.5
C11—N10—H10	114.2 (9)	O20—C21—H21C	109.5
C12—C11—C16	118.65 (12)	H21A—C21—H21C	109.5
C12—C11—N10	116.99 (11)	H21B—C21—H21C	109.5
C16—C11—N10	124.31 (12)	C5—O22—C23	118.12 (12)
C13—C12—C11	120.76 (12)	O22—C23—H23A	109.5
C13—C12—H12	119.6	O22—C23—H23B	109.5
C11—C12—H12	119.6	H23A—C23—H23B	109.5
C12—C13—C14	121.44 (13)	O22—C23—H23C	109.5
C12—C13—H13	119.3	H23A—C23—H23C	109.5
C14—C13—H13	119.3	H23B—C23—H23C	109.5

### Hydrogen-bond geometry (Å, °)

**Table:**

*D*—H···*A*	*D*—H	H···*A*	*D*···*A*	*D*—H···*A*
N7—H7···O20	0.865 (15)	2.227 (14)	2.6113 (16)	106.7 (11)
O19—H19···O9^i^	0.867 (18)	1.841 (19)	2.7080 (14)	178.0 (17)
N10—H10···O19^ii^	0.867 (14)	2.161 (14)	2.9799 (15)	157.4 (12)
N7—H7···O19^ii^	0.865 (15)	2.189 (15)	2.9837 (15)	152.6 (13)

Symmetry codes: (i) −*x*+1, *y*+1/2, −*z*+3/2; (ii) −*x*+1, −*y*+1, −*z*+1.
